# Three-Dimensional Assessment of the Biological Periacetabular Defect Reconstruction in an Ovine Animal Model: A µ-CT Analysis

**DOI:** 10.3390/bioengineering12070729

**Published:** 2025-07-03

**Authors:** Frank Sebastian Fröschen, Thomas Martin Randau, El-Mustapha Haddouti, Jacques Dominik Müller-Broich, Frank Alexander Schildberg, Werner Götz, Dominik John, Susanne Reimann, Dieter Christian Wirtz, Sascha Gravius

**Affiliations:** 1 Department of Orthopaedics and Trauma Surgery, University Hospital Bonn, 53127 Bonn, Germanyfrank.schildberg@ukbonn.de (F.A.S.); sascha.gravius@umm.de (S.G.); 2 Augustinian Sisters Hospital, Clinic for Orthopedics, Special Orthopedic Surgery and Sports Medicine, 51109 Cologne, Germany; 3 Independent Researcher, 53125 Bonn, Germany; 4 Department of Oral Medical Technology, School of Dentistry, University of Bonn, 53127 Bonn, Germany; 5 Practice for Orthopaedics and Sports Traumatology, 53115 Bonn, Germany; 6 Department of Medical Engineering, University of Applied Sciences Bremerhaven, 27568 Bremerhaven, Germany; 7 Orthopaedic and Trauma Surgery Centre, University Hospital Mannheim, Medical Faculty Mannheim of the University of Heidelberg, 68167 Mannheim, Germany

**Keywords:** acetabular bone defect, animal model, µ-CT, arthroplasty, hip, revision total hip arthroplasty

## Abstract

The increasing number of acetabular revision total hip arthroplasties requires the evaluation of alternative materials in addition to established standards using a defined animal experimental defect that replicates the human acetabular revision situation as closely as possible. Defined bone defects in the load-bearing area of the acetabulum were augmented with various materials in an ovine periacetabular defect model (Group 1: NanoBone^®^ (artificial hydroxyapatite-silicate composite; Artoss GmbH, Germany); Group 2: autologous sheep cancellous bone; Group 3: Tutoplast^®^ (processed allogeneic sheep cancellous bone; Tutogen Medical GmbH, Germany)) and bridged with an acetabular reinforcement ring of the Ganz type. Eight months after implantation, a μ-CT examination (*n* = 8 animals per group) was performed. A μ-CT analysis of the contralateral acetabula (*n* = 8, randomly selected from all three groups) served as the control group. In a defined volume of interest (VOI), bone volume (BV), mineral volume (MV), and bone substitute volume (BSV), as well as the bone surface (BS) relative to the total volume (TV) and the surface-to-volume ratio (BS/BV), were determined. To assess the bony microarchitecture, trabecular thickness (Tb.Th), trabecular separation (Tb.Sp), and trabecular number (Tb.N), as well as connectivity density (Conn.D), the degree of anisotropy (DA), and the structure model index (SMI), were evaluated. The highest BV was observed for NanoBone^®^ (Group 1), which also showed the highest proportion of residual bone substitute material in the defect. This resulted in a significant increase in BV/TV with a significant decrease in BS/BV. The assessment of the microstructure for Groups 2 and 3 compared to Group 1 showed a clear approximation of Tb.Th, Tb.Sp, Tb.N, and Conn.D to the microstructure of the control group. The SMI showed a significant decrease in Group 1. All materials demonstrated their suitability by supporting biological defect reconstruction. NanoBone^®^ showed the highest rate of new bone formation; however, the microarchitecture indicated more advanced bone remodeling and an approximate restoration of the trabecular structure for both autologous and allogeneic Tutoplast^®^ cancellous bone when using the impaction bone grafting technique.

## 1. Introduction

To date, the biological downsizing of periacetabular bone defects with autologous cancellous bone remains the gold standard in revision total hip arthroplasty (RTHA) [1,2,3]. Nevertheless, the amount of autologous cancellous bone available may be limited in RTHA, often requiring a second surgical approach for harvesting. Previous studies have reported a complication rate (bleeding, postoperative infection, persistent pain) at the donor site of up to 30% [4,5]. Nevertheless, bone defect reconstruction with autologous cancellous bone continues to be the “gold standard” in terms of a biological downsizing prior to defect reconstruction [1,6,7].

Today, different bone substitute materials are available, which differ in composition and structure. In general, the available materials are used in clinical practice in various fields and can be divided into five categories: materials of natural origin, synthetic materials, composite materials, materials combined with growth factors, and materials loaded with living cells [8].

An already well-known bone substitute material in dental and maxillofacial surgery is NanoBone^®^. NanoBone^®^ is a synthetic material consisting of nanocrystalline hydroxyapatite embedded in a highly porous silica gel matrix, providing a very large surface area [9].

Previous studies evaluating the properties of NanoBone^®^ have reported limited resorbability, although the material demonstrates high biocompatibility, free availability, osteoconductivity, and no immunogenic potential [9,10].

Nevertheless, the number of studies evaluating the use of synthetic bone substitute materials in RTHA under load-bearing conditions is limited [11,12]. As this question cannot be answered in the context of a clinical study on humans for ethical reasons, an animal model might be helpful.

In detail, evaluation under prospective conditions using a µ-computed tomography scan after surgery in an ovine animal model might be a promising solution, as sheep are not able to fully avoid load-bearing of the hip, while still allowing the opportunity for free-range husbandry under the supervision of a shepherd [13,14,15,16,17]. The main advantage of the µ-CT scan is its ability to provide non-destructive determination of volumetric and geometric parameters of the evaluated bone stock [18,19].

This study was designed to characterize, compare, and evaluate the biological bone defect reconstruction with the synthetic NanoBone^®^ bone substitute material in comparison to Tutoplast-cancellous bone^®^ as a representative of well-established allogenic cancellous bone, and autologous cancellous bone as the “gold standard” in the load-bearing area of the acetabulum after the artificial creation of a critical size bone defect according to Aaron and Skerry [20].

Our hypothesis was that reconstruction with artificial or allogenic materials in the animal model sheep is not inferior to reconstruction with autologous cancellous bone in the load-bearing area of the acetabulum.

## 2. Materials and Methods

The study was approved by the official state animal care and use committee (LANUV NRW, 8.87-50.10.35.08.308). All experiments were performed in accordance with German federal law regarding the protection of animals, institutional guidelines, and the criteria set forth in the “Guide for the Care and Use of Laboratory Animals” (National Institutes of Health [Publication, 8th Edition] 2011).

### 2.1. Animal Model and Surgical Technique

As described in our previous publication, a defined bone defect of 3.375 cm^3^ (1.5 × 1.5 × 1.5 cm) in the cranial load-bearing area of the acetabulum was established and subsequently augmented with two different biomaterials as well as autologous cancellous bone. Subsequently, the defect was bridged with a Ganz reinforcement ring (ARR; Brehm GmbH, Weisendorf, Germany) before implantation of a cemented cup. A cemented monobloc femoral implant made of CoCrMo was used (Aesculap, Tuttlingen, Germany). For implantation, we performed the same surgical technique that is used in humans after augmentation of defined bone defects in the load-bearing area of the acetabulum. All surgeries were performed on the left hip joint using the described surgical technique [21].

#### 2.1.1. Group Setup

We defined 3 groups with 9 animals each based on the material used for the reconstruction of the bony defect:

Group 1: NanoBone^®^ (Artoss GmbH, Rostock, Germany), as a representative of the hydroxyapatite-silicate composites, is produced in the low temperature range (<700 °C) using the sol–gel process [22,23]. The crystal size of the hydroxyapatite contained in NanoBone^®^ corresponds roughly to that in human bone [24]. Hydroxyapatite is pulverized and placed in a SiO_2_ sol (based on alkoxides) and distributed homogeneously. During the gel transition, a nanoporous basic structure is formed via SiO_2_ bonds, which connect the loosely packed hydroxyapatite crystals to each other. As the solvent vaporizes during the drying process at a maximum of 200 °C, shrinkage and micropores of 5–100 µm are formed. During the crystallization process, an interconnecting pore system with a size of 10–20 nm develops in silica gel (SiO_2_) [25,26]. The result is a highly porous shaped body with a solids density of 0.5–0.7 g/cm^3^, which corresponds to a degree of porosity of 60–80%, while maintaining a relatively high mechanical pressure resistance of approximately 40 MPa [22,25]. NanoBone^®^ was used as a NanoBone^®^ block (dimensions 5 × 10 × 15 mm) and as rough granules (1 × 2 mm).

Group 2: “Impaction-Bone-Grafting” with autologous sheep cancellous bone. This was obtained from the previously resected femoral heads of the operated sheep. The femoral heads were completely cleared of cartilage, and bone chip autografts measuring approximately 2–5 mm^3^ were produced using a rongeur forceps. Processing with a heat-generating saw was avoided. To increase the mechanical strength, blood, fat, and medullary parts were washed out of the autologous bone chips.

Group 3: “Impaction-Bone-Grafting” with allogeneic ovine cancellous bone chips processed according to the Tutoplast^®^ method (Tutogen Medical GmbH, Neunkirchen am Brand, Germany). The Tutoplast^®^ cancellous bone was prepared from femoral heads of the same breed of sheep by the Tutoplast process (Tutogen^®^ Medical GmbH, Neunkirchen am Brand, Germany). Processing was carried out according to established methods for the production of human Tutoplast^®^ cancellous bone. The processed femoral heads were divided into bone chips measuring approximately 2–5 mm^3^ under sterile conditions and were then rehydrated according to the manufacturer’s instructions (Tutogen^®^ Medical GmbH, Neunkirchen am Brand, Germany).

Control group: We performed a μ-CT analysis of the contralateral native sheep acetabula (*n* = 8) to visualize the ovine acetabular microarchitecture. The sheep were randomly selected from all three groups. This facilitates the definition of the final state after complete bone remodelling and resorption of the implanted bone substitute material. For evaluation and interpretation of the µ-CT results, this was defined as the control group.

#### 2.1.2. Sample Retrieval and Preparation

**Figure 1:**
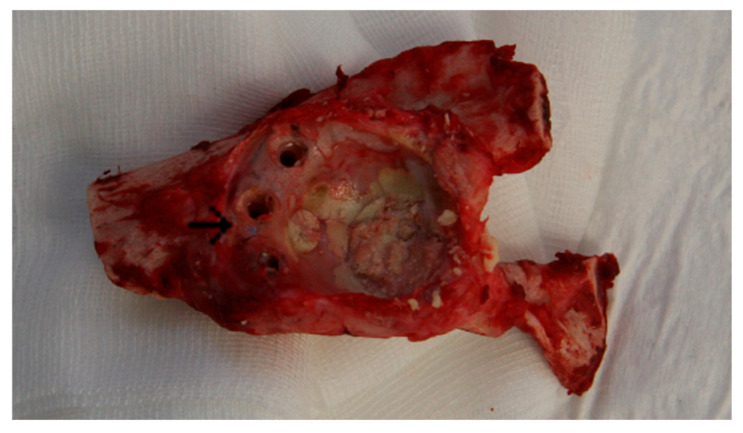
Acetabulum following removal of the polyethylene cup, cement fixation, and explantation of the Ganz reinforcement ring as well as the rim screws; the central rim screw (→) served as a reference structure to identify the location of the former defect area.

Eight months after implantation, euthanasia was carried out immediately after sedation of the sheep by the rapid application of embutramide (T61, Intervet Deutschland GmbH, Unterschleißheim, Germany) via an intravenous catheter in an overdose of 10–15 mL. Before µ-CT evaluation, the inlying implants were explanted to allow the best possible assessment (Figure 1).

### 2.2. µ-CT Evaluation

The μ-CT analysis of the augmented defect areas was performed using the SkyScan 1174 μ-computed tomography scanner (SkyScan, Kontich, Germany). For the fully automated assessment of the volume proportions of different tissues and various bone structural parameters, a volume of interest (VOI) was defined within the original boundaries of the augmented defects by analyzing the reconstructed volume datasets. To avoid negative influences on the analysis, such as artifacts on the outer surfaces of the samples, a central positioning of the VOI within the augmented defects (15 × 15 × 15 mm) was required. For this purpose, a cube measuring 5 × 5 × 5 mm (VOI 125 mm^3^) was placed centrally within the predefined defect area, ensuring that the evaluated VOI—including the surfaces—remained safely within the defect.

The VOI was deliberately chosen in the center of the defect, because here—at a sufficient distance from the interface with the native bone—the bone remodelling and the capacity of the tested materials could be assessed in a representative and standardized manner. Specifically, the total volume (total volume [TV]), the volume of the mineralized tissue (mineral volume [MV]), the volume of the newly formed bone (bone volume [BV]), and the remaining bone substitute material (bone substitute volume [BSV]) were determined.

The microstructural parameters were recorded by the analysis software NRecon^®^ V1. from Scanco Medical AG (Wangen-Brütisellen, Switzerland).

The microstructural parameters were used to consistently describe and quantify the trabecular structure of the bone [27].

The following parameters were recorded in a standardized manner:

Bone volume fraction (BV/TV) [%];

Mean trabecular thickness (Tb.Th) [μm];

Mean trabecular separation (Tb.Sp) [μm];

Mean trabecular number (Tb.N) [1/cm];

Connectivity density (Conn. D) [1/mm^3^];

Geometric degree of anisotropy (DA);

Structure model index (SMI);

Bone mineral density (BMD).

### 2.3. Statistical Analysis

The values obtained from the investigations were converted and exported to MS Excel (Microsoft, Redmond, WA, USA) and SPSS 20 (SPSS IBM Statistics, IBM, New York, NY, USA). Continuous variables were represented by means ± standard errors and the indication of minimum and maximum, categorical variables by the indication of absolute and relative frequencies.

The data obtained were subjected to a one-factorial or multifactorial variance analysis with a subsequent Bonferroni procedure as a post hoc test. An alpha of 0.05 was considered significant. All variables were tested for the homogeneity of the variances using Levene statistics.

## 3. Results

### 3.1. Animal Experiment: Macroscopic Assessment

During the course of the experiment, three postoperative complications occurred before the end of the observation period, which led to the termination of the animal experiment in those cases (one complication per group—Group 1: cervical abscess with septic circulatory instability 22 days postoperatively; Group 2: periprosthetic fracture of the femur 10 days postoperatively; Group 3: pneumonia 21 days postoperatively). This resulted in a final number of n = 8 animals per group, which were available for test evaluation after an observation period of 8 months. After sample retrieval, µ-CT analysis was successfully performed in all cases.

### 3.2. Qualitative Marker Assessment

#### 3.2.1. NanoBone^®^

**Figure 2:**
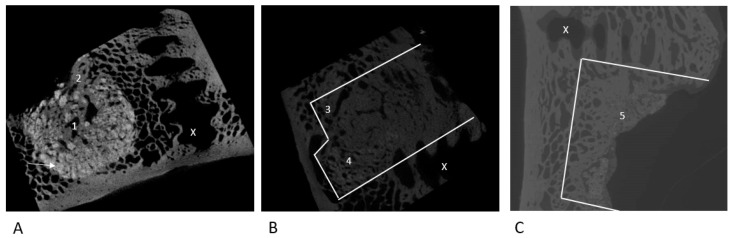
Exemplary μ-CT images of the NanoBone^®^ group (**A**), Tutoplast group (**B**) and cancellous bone group (**C**); (→: residual radiopaque NanoBone^®^. (1) In the center and at the limits (2) of the defect, advanced resorption with partial restoration of the trabecular bone structure; (3) almost total reconstruction of native bone structure; (4) residual Tutoplast-cancellous bone; (5) almost total reconstruction of the trabecular structure with near complete resorption of the autologous cancellous bone; (X) screw hole. White lines highlight the defect area).

The μ-CT images showed NanoBone^®^ as a radiopaque bone substitute material in the augmented defect area (Figure 2A). The former defect zones could be clearly distinguished from the surrounding native bone stock. Trabecular structures penetrating the augmented area and surrounding the remaining NanoBone^®^ could be detected in all cases. In some samples—especially at the interface with the native bone but also centrally within the periacetabular defects—an advanced remodeling with indicated reconstruction of the physiological cancellous structure could be observed. Nevertheless, densely packed NanoBone^®^ residues were found in all cases adjacent to the cortex on the inner side of the pelvis.

#### 3.2.2. Autologous Sheep Cancellous Bone

In all cases in Group 2, we were able to detect an almost complete resorption of the autologous bone graft with near-complete restoration of the trabecular bone structure within the defect area (Figure 2C). There was almost no graft material detectable within the defect anymore. The newly formed bone stock showed a denser trabecular bone structure.

#### 3.2.3. Tutoplast^®^ Cancellous Bone

In all cases, an advanced resorption of the allogeneic Tutoplast^®^ cancellous bone with formation of new bone stock could be detected (Figure 2B). A restoration of the trabecular bone structure could be documented in the marginal areas of the defect. Tutoplast^®^ residues were sometimes integrated into the trabecular structure and surrounded by newly formed bone. In the center, there were areas of tightly packed, newly formed bone with only isolated Tutoplast^®^ residues. Macroscopically, we could detect—starting from the autochthonous bone stock—newly formed bone and advancing towards the center of the artificial defect. Overall, newly formed bone was present throughout the entire defect area.

### 3.3. MV/TV; BV/TV; BSV/TV; BS/TV

**Figure 3:**
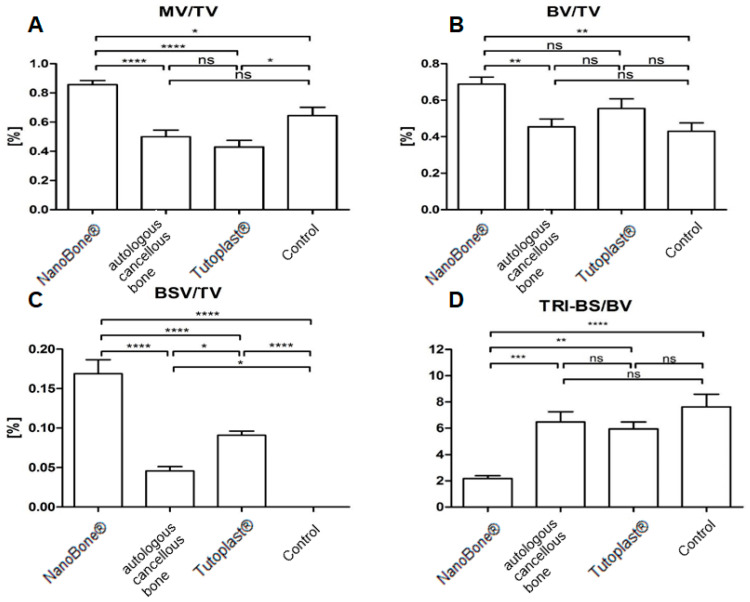
Statistical analysis for NanoBone^®^, Tutoplast^®^, autologous cancellous bone and the control group with display of the statistical evaluation for MV/TV [%], BV/TV [%], BSV/TV [%] and bone surface to volume ratio of bone (BS/BV) [mm^−1^] ((**A**–**D**); ns = not significant; * = *p* < 0.05; ** = *p* < 0.01; *** = *p* < 0.001; **** = *p* < 0.0001).

The individual results and the associated levels of significance are shown in Table 1 and Figure 3A–D. NanoBone^®^ displayed the highest ratio of MV/TV and BV/TV in comparison to the control group, while showing a significantly higher BSV/TV in comparison to the Tutoplast^®^ and autologous bone groups.

### 3.4. Tb.Th, Tb.Sp, and Tb.N

**Figure 4:**
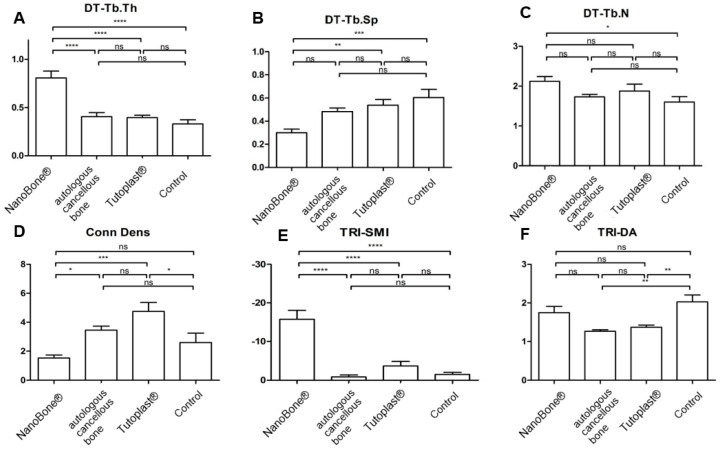
Statistical analysis for trabecular thickness [Tb.th], trabecular separation [Tb.sp], trabecular number [Tb.N], connectivity density [Conn. D], the structure model index [SMI], and the degree of anisotropy [DA] for all groups with display of the statistical evaluation ((**A**–**F**); ns = not significant; * = *p* < 0.05; ** = *p* < 0.01; *** = *p* < 0.001; **** = *p* < 0.0001).

Table 1 provides the descriptive summary of the assessment of the microstructure for all groups. Figure 4 displays the statistical analysis for the four groups. For Tb.Th, the NanoBone^®^ had a significantly higher result in comparison to all other groups (*p* ≤ 0.05). For Th.SP, the control group showed the highest value with a significant difference compared to the NanoBone^®^. For Tb.N, the NanoBone^®^ group showed the highest values, with a significant difference compared to the control group (*p* ≤ 0.05).

### 3.5. Conn.D; SMI; DA

The results for the connectivity density (Conn.D) as a value for the interlinking of trabecular structure, the structure model index (SMI) as a value for the macroscopic structure of the trabecular bone, and the degree of anisotropy (DA) describing the three-dimensional structure of the bone are shown in Table 1. Tutoplast^®^ displayed the highest values with a significant difference compared to the NanoBone^®^ group for Conn.D and SMI (Figure 3). In contrast, the SMI showed the lowest values in the NanoBone^®^ group. The highest values for the geometric degree of anisotropy (DA) were recorded in the control group. The individual results and the associated level of significance are shown in Table 1 and Figure 4.

### 3.6. BMD

**Figure 5:**
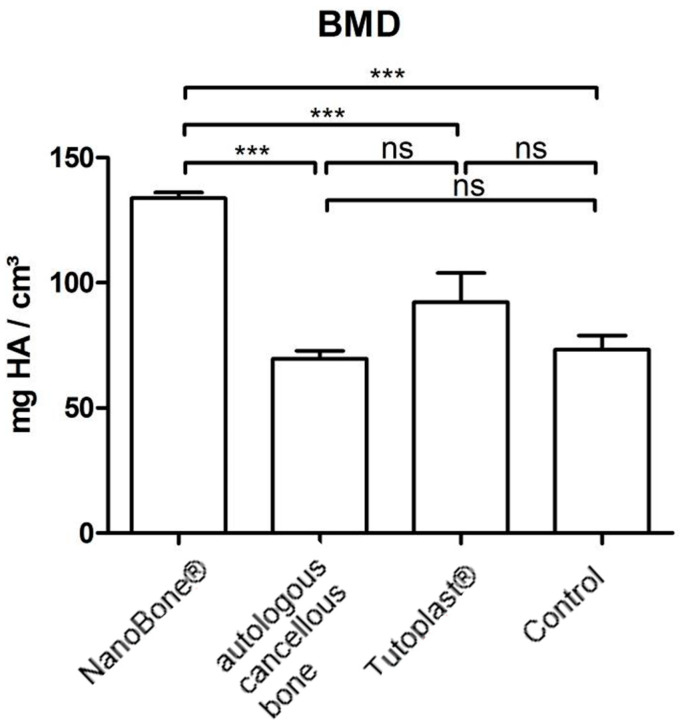
Statistical analysis for NanoBone^®^, Tutoplast^®^, autologous cancellous bone, and the control group for bone mineral density (BMD) [mg HA/cm^3^] (ns = not significant; *** = *p* < 0.001;).

The highest BMD could be found in the NanoBone^®^ group, followed by the Tutoplast^®^ group, the autologous bone group, and finally the control group. The individual results can be found in Figure 5 and Table 1.

## 4. Discussion

Our main finding was that all the different bone substitute materials used were able to demonstrate their suitability for acetabular defect reconstruction in the sheep animal model.

This is crucial as long-term treatment success is dependent on successful periacetabular bone defect reconstruction. Nevertheless, the quality of the remodelled bone is a relevant parameter. In this context, Jiang et al. defined volumetric and geometric parameters for evaluation, while structural parameters such as Tb.Th, Tb.Sp, and Tb.N were used to evaluate the degree of new bone formation [18,28,29].

The BV/TV showed significant differences between the NanoBone^®^ group and both the autologous cancellous group and the control group after a standing time of 8 months. This correlated with a significantly increased Tb.Th for the NanoBone^®^ group compared to all other groups. Similarly, the BMD showed the highest values for the NanoBone^®^ group with significant differences compared to the other bone substitute materials. The evaluations suggest that the increase in BV/TV of the NanoBone^®^ group resulted primarily from the increase in trabecular thickness (Tb.Th) and not the trabecular number (Tb.N) or the trabecular distance (Tb.Sp). Nevertheless, the high residual material volume in the NanoBone^®^ group might affect the evaluation of parameters such as BV/TV and MV/TV. In addition, a high BV/TV but, in contrast, a low BS/BV might suggest a poor trabecular connectivity. BS/BV remains an important structural parameter: a high BS/BV indicates a greater surface area per unit of bone volume, which promotes osteoblast attachment, cell migration, nutrient exchange, and vascular ingrowth. In contrast, a low BS/BV indicates a more compact bone structure, which impedes cellular infiltration, limits vascularization, and subsequently hampers bone regeneration [30,31]. As BS/BV in the NanoBone^®^ group was significantly lower compared to the other materials at 8 months standing time, long-term results are needed.

Tb.N showed, similar to Tb.Th, the highest number of trabeculae for the NanoBone^®^ group—but without any significant differences to Tutoplast^®^ or the autologous cancellous bone. In contrast, NanoBone^®^ displayed the lowest trabecular distance (Th.SP) with significant differences from Tutoplast^®^.

If the µ-CT analysis of the native acetabular samples is used and their microarchitecture is simply defined as the “ideal” final state after complete bone remodeling, the autologous cancellous bone, as well as Tutoplast^®^, did not display any significant differences for Tb.Th, Tb.N, Tb.Sp, BV/TV, and BS/TV. The differences in BV/TV between the autologous cancellous bone group (45.4 ± 4.3%) and the control group (42.9 ± 4.6%) were not significant, but they highlight that in animal experiments with a low number of animals, further studies might be helpful for detailed evaluation. According to Schmidmeier et al., BV/TV can return to levels comparable to native bone within 8–12 weeks [32].

In contrast, NanoBone^®^ showed significant differences for those parameters. Therefore, it might be possible that bone remodeling in these two groups might be further advanced. These hypothesis is supported by Conn.D and SMI as markers for the trabecular bone structure [33]. A high Conn.D is associated with the formation of a new dense trabecular network, while in the course of a reorganization of the trabecular structures (“bone remodeling”) through osteoclastic remodeling, a reduction in the Conn.D can be observed [29,34].

A possible explanation for the significantly lower Conn.D results for our NanoBone^®^ group might be ongoing bone remodeling 8 months after surgery or increased osteoclast activity due to continued resorption of the artificial material. The latter is supported by the amount of NanoBone^®^ within the defect [34]. Nevertheless, NanoBone^®^ showed the highest bone density and BV/TV, indicating sustained new bone formation. It should be noted that the value of Conn.D as a parameter for the connectivity of the trabecular structures is still unclear [35]. Conn.D only measured quantity but not quality; therefore, the orientation, structure, and strength of the trabecular connection are also relevant [36,37]. Previous studies could outline in this context a reduced bone strength, despite a high Conn.D, since Conn.D does not evaluate the quality of the trabecular connection [38]. In this context, the SMI might be a relevant parameter. The domain of the SMI is its ability to distinguish between samples of equal bone density, but different trabecular structure [18,39]. Although the SMI of the autologous cancellous bone group and the Tutoplast^®^ group approximated the values of the native acetabular samples, the SMI for the NanoBone^®^ group was clearly negative. A possible explanation might be the remaining artificial bone substitute material. In addition, the NanoBone^®^ group displayed the highest geometric degree of anisotropy (DA) as an indicator of a three-dimensional trabecular orientation of the trabecular structures and a parameter for the mechanical properties of the bone without any significant difference to the autologous cancellous bone or Tutoplast^®^ groups. Nevertheless, the limitations of Conn.D should not be overemphasized. The lower Conn.D in the NanoBone^®^ group indicates fewer trabecular connections per unit volume, which may result in a less integrated and mechanically weaker trabecular network. Thus, a significantly reduced Conn.D in the NanoBone^®^ group at 8 months standing time could imply that the regenerated bone structure at this point lacks the necessary interconnections to mimic the biomechanical competence of native bone. The feasibility of using NanoBone^®^ in RTHA is of considerable clinical importance, as such procedures often involve large, load-bearing bone defects that require robust and durable bone regeneration. Here the observed lower Conn.D compared to the other groups suggest that a reconstructed defect with NanoBone^®^ may be mechanically inferior. This might be critical in the load-bearing region of the acetabulum. Evaluation after a longer standing time might be helpful to evaluate or exclude a suspected aseptic loosening. In contrast, Götz et al. and Yamada et al. evaluated the role of bone defect reconstruction with NanoBone^®^ in non-load-bearing areas with satisfying results [9,40]. Nevertheless, other synthetic alternatives—such as HA scaffolds—fail to replicate the microstructure required for mechanical load-bearing in large defects compared to autografts or allografts too [41].

In general, the remaining NanoBone^®^ material might influence bone remodeling by favoring a macrophage polarization towards reparative M2 phenotype, decreasing the pro-inflammatory M1 population, and ensuring a pro-regenerative response at the implantation site of these biomaterials, thereby promoting bone repair [42]. Therefore, long-term evaluation might generate additional information.

Based on our observations regarding Conn.D and BS/BV, future developments should focus on improving the micro-structure to better mimic the native architecture. Here, the control and optimization of the resorption kinetics with a balance between degradation and mechanical stability during the early phases of bone formation is essential. Advanced manufacturing techniques such as 3D printing might be a possible solution [43].

Our study has limitations, the most relevant being the limited evaluation time and the small number of animals. In particular, the limited sample size might have a relevant influence on the µCT evaluation. Here, we acknowledge that economic aspects in combination with the approved protocol always play a role in large animal models. Including an additional group with a defined bone defect but without subsequent reconstruction was not possible for ethical reasons. Studies with a larger number of animals and a longer postoperative evaluation period might provide additional information. Nevertheless, the presented data might still be useful as they give an impression of the progress of bone defect reconstruction at a certain time point.

In summary, among all materials tested, NanoBone^®^ had the highest rate of new bone formation. The microarchitecture indicated an “advanced” bone remodelling for NanoBone^®^, with an almost complete restoration of the trabecular structure for the autologous and allogeneic Tutoplast^®^ cancellous bone using the impaction bone grafting technique. Our results confirm that the autologous cancellous bone must still be regarded as the gold standard in the treatment of bony defects, while Tutoplast^®^ and NanoBone^®^ are viable alternatives to promote osteogenesis.

## Figures and Tables

**Table 1:** Descriptive summary (mean ± standard deviation) of the µ-CT scan results for all three used materials for defect reconstruction [MV/TV: mineral volume; BV/TV: bone volume; BSV/TV: bone substitute volume; BS/BV: bone surface per bone volume ratio; TV: total volume; Tb.Th: trabecular thickness (measure of the thickness of the trabeculae in the total volume); Th.SP: trabecular separation (mean distance between the trabeculae within the sample volume); Tb.N: trabecular number (number of trabecular within the analyzed volume); Conn. D: connectivity density [1/mm^3^]; SMI: structure model index; DA: geometric degree of anisotropy; BMD: bone mineral density].

	NanoBone^®^	Autologous Cancellous Bone	Tutoplast^®^	Control Group
MV/TV [%]	85.74 ± 2.69	49.97 ± 4.54	64.49 ± 5.63	42.94 ± 4.6
BV/TV [%]	68.85 ± 3.76	45.4 ± 4.3	55.42 ± 5.33	42.94 ± 4.6
BSV/TV [%]	16.9 ± 1.75	4.57 ± 0.55	9.07 ± 0.53	
BS/BV [mm^−1^]	2.17 ± 0.22	6.47 ± 0.78	5.95 ± 0.53	7.62 ± 0.96
Tb.Th [µm]	0.81 ± 0.07	0.39 ± 0.05	0.39 ± 0.02	0.33 ± 0.04
Th.SP [µm]	0.3 ± 0.03	0.48 ± 0.03	0.54 ± 0.05	0.6 ± 0.07
Tb.N [1/cm]	2.12 ± 0.12	1.73 ± 0.06	1.88 ± 0.17	1.6 ± 0.13
Conn. D [1/mm^3^]	1.53 ± 0.21	3.46 ± 0.27	4.75 ± 0.62	2.6 ± 0.66
SMI	−15.77 ± 2.29	−0.86 ± 0.49	−3.65 ± 1.19	−1.51 ± 0.54
DA	1.75 ± 0.45	1.27 ± 0.11	1.37 ± 0.16	2.03 ± 0.5
BMD [mg HA/cm^3^)	133.78 ± 6.55	73.32 ± 15.81	92.32 ± 32.92	69.68 ± 8.8

## Data Availability

The original contributions presented in the study are included in the article; further inquiries can be directed to the corresponding author.
